# Comparison of HIV prevention indicators among adolescent girls and young women in DREAMS and non-DREAMS intervention districts in Uganda

**DOI:** 10.1371/journal.pone.0321277

**Published:** 2025-04-01

**Authors:** Norah Namuwenge, Derrick Kimuli, Rebecca N. Nsubuga, Timothy Sserunga, Sheila Nyakwezi, Jaffer Byawaka, Garoma Kena, Solome Sevume, Norbert Mubiru, Barbara Amuron, Daraus Bukenya

**Affiliations:** 1 Social & Scientific Systems, a DLH Holdings company, United States Agency for International Development Strategic Information Technical Support Activity, Kampala, Uganda; 2 The United States Agency for International Development Uganda, US Mission Compound - South Wing, Kampala, Uganda; Johns Hopkins University Bloomberg School of Public Health, UNITED STATES OF AMERICA

## Abstract

In sub-Saharan Africa, a significant number of new human immunodeficiency virus (HIV) infections occur among adolescent girls and young women (AGYW). The 2023 Uganda Annual Spectrum estimates indicated that about one-third of all new HIV infections are among AGYW. In 2016, the Ministry of Health in partnership with the United States President’s Emergency Plan for AIDS Relief (PEPFAR) initiated the Determined, Resilient, Empowered, AIDS-Free, Mentored and Safe (DREAMS) program to reduce the vulnerability of AGYW to HIV by offering various direct and indirect HIV-related prevention services. These services influence the level of various HIV prevention indicators in the age group. This study aimed to compare these levels. The study was a secondary analysis of pooled Lot Quality Assurance Sampling (LQAS) survey data collected in DREAMS and non-DREAMS districts during 2021 and 2022. Fifteen HIV prevention indicators were independently compared between 8 DREAMS and 8 non-DREAMS districts. Chi-square tests were used to assess the significance in the differences. Of the 9,290 records of AGYW reviewed, 52.40% were of AGYW residing in DREAMS districts. Between DREAMS and non-DREAMS districts, significant differences in level of knowledge of HIV prevention methods (25.60% versus 14.63%, *p* < 0.001), condom use (58.99% versus 48.33%, *p* < 0.001), knowledge of HIV testing points (93.43% versus 92.38%, *p* = 0.049), having multiple sex partners (15.28% versus 10.11%, *p* < 0.001), condom use (58.99% versus 48.33%, *p* < 0.001), HIV testing (84.86% versus 82.00%, *p* < 0.001) and multiple sex partners (15.28% versus 10.11%, *p* < 0.001) among other indictors. AGYW in DREAMS districts had better outcomes for all indicators except multiple sex partners. Although this factor likely contributed to the initial selection of DREAMS-intervention districts, its persistence may continue to influence overall efforts towards the reduction of HIV prevalence. Moreover, this potentially mitigates the benefits from other better performing indicators.

## Introduction

Adolescent girls and young women (AGYW) in sub-Saharan Africa (SSA) are disproportionately affected by human immunodeficiency virus (HIV). AGYW account for 80% of new HIV infections among adolescents with an estimated 460 of new infections and 50 AIDS-related deaths occurring daily in this group [1]. In Uganda, the HIV incidence among girls aged 15–24 remains significantly higher than that of their male counterparts and women in older age groups [2]. As a response, the President’s Emergency Plan for AIDS Relief (PEPFAR) launched the Determined, Resilient, Empowered, Acquired immunodeficiency syndrome (AIDS)-Free, Mentored, and Safe (DREAMS) program, a multi-component initiative designed to reduce HIV infections and improve the overall well-being of AGYW [3,4]. Since its implementation in Uganda in 2016, DREAMS has provided a package of evidence-based interventions that address the underlying factors contributing to HIV risk among AGYW. DREAMS interventions support AGYW through providing education and economic strengthening, reducing HIV risk through HIV testing and prevention services, mobilizing communities to shift gender norms and reduce gender-based violence (GBV), and strengthening families through social protection initiatives.

Regionally, studies from Kenya and South Africa have shown mixed results regarding the benefits of DREAMS. While DREAMS interventions in these countries had limited influence on school retention and reducing GBV, they significantly increased HIV status awareness among AGYW and reduced risk behaviors in rural Kenya [5–7]. In Lesotho, there was a notable reduction in new HIV diagnoses among AGYW when comparing DREAMS and non-DREAMS districts [8]. Studies in Uganda have highlighted high participation rates in DREAMS interventions (when offered) and significant reduction HIV risk behaviors among [9,10]. Another reported benefits such as improved HIV testing and condom use [11].

As Uganda and SSA strive to end the HIV/AIDS epidemic, the need for data-driven insights into key interventions like DREAMS is increasingly clear [12,13]. The efforts of DREAMS interventions is in Uganda can measured through indicators related to education, HIV prevention, and sexual risk behaviors. For instance marital status is a proxy for early marriage, which is associated with an increased HIV risk [14]; school enrollment can be protective against HIV as it improves awareness and delays sexual debut [15]; HIV testing, status disclosure, HIV knowledge, and partner testing – along with sexual behavior indicators such as sexual activity, condom use, and number of sexual partners - are direct or indirect indicators of HIV prevention [4]. Given that previous studies were mostly qualitative and limited in geographic and indicator coverage in addition to being non-comparable, this study addresses the gap. The study leverages the Lot Quality Assurance Sampling (LQAS) survey conducted in both DREAMS and non-DREAMS districts in Uganda to compare performance [16–18]. It assessed the status in DREAMS districts by examining whether AGYW in DREAMS-supported areas demonstrates better levels of HIV prevention indicator outcomes compared to their counterparts in non-DREAMS districts. The study helps to demonstrate efforts and gaps thereby generating additional evidence to guide future HIV prevention strategies among AGYW in Uganda and similar contexts.

## Materials and methods

### DREAMS in Uganda

With prevalence rates (shown in brackets) higher than those in neighboring districts, Agago (6.2%), Gulu (12.4%), Omoro (6.3%), Oyam (6.3%), Apac (2.3%), Kwania (6.5%), Lira (7.2%), and Mbarara (13.1%) face considerable challenges in controlling HIV, [19]. In Northern Uganda, districts like Gulu, Agago, Omoro, Oyam, and Lira have a history of conflict, which has led to social disruption, poverty, and limited access to healthcare, exacerbating HIV transmission risks among vulnerable populations, particularly adolescent girls and young women [2,20,21]. Apac and Kwania in the mid-northern region have similarly struggled with high HIV rates due to limited healthcare infrastructure and economic challenges that push some residents into high-risk behaviors. In Western Uganda, Mbarara stands out with higher HIV prevalence partly due to its role as a regional hub, drawing in diverse populations and increasing the risk of transmission [22]. In all these districts, efforts focus on prevention, treatment, and community outreach to address the socio-economic drivers of HIV and improve access to healthcare services. Initially launched in 10 districts, the DREAMS program was expanded to 23 districts in Uganda, targeting areas with high HIV prevalence. DREAMS encompasses a holistic, multi-level approach targeting AGYW aged 10–24 years with tailored interventions based on their age group and risk profile. The strategy includes primary, secondary, and contextual services that cater to the unique needs of AGYW, their partners, and the broader community.

The primary package of services is age-specific and focuses on HIV prevention, violence prevention, mental health, social asset building, and socio-economic support. For younger AGYW (10–14 years), the focus is on community-based HIV and violence prevention programs like “No Means No,” while older AGYW (15–19 years and 20–24 years) receive additional services such as sexually transmitted infections (STI) screening, Pre-Exposure Prophylaxis (PrEP) screening, and socio-economic approaches for out-of-school AGYW. The primary services must be completed for an AGYW to graduate from the program. Secondary services are provided based on specific risk factors identified through baseline and follow-up assessments. These services include HIV testing, STI treatment, gender-based violence (GBV) screening, post-violence care, and socio-economic support like short-term trade skills and asset financing. Contextual services target the broader community and AGYW’s sexual partners to create a supportive environment for HIV prevention. These services include violence prevention for adolescent boys and young men, reducing risk in sexual partners through HIV testing services (HTS), PrEP, voluntary medical male circumcision (VMMC), and antiretroviral therapy (ART), and community mobilization efforts.

### About the LQAS survey

For more detailed information on the LQAS survey in Uganda, interested parties can refer to the referenced here [16,18,23–25]. However, in summary, the LQAS survey is a multi-indicator health survey with an approach that involved the division of a district into 5–7 supervision units based on established criteria such as administrative boundaries and population attributes. Probability proportional to size sampling technique was then used to select either 19 or 24 villages from each supervision unit. At the village level, a reference household for the survey was determined using random sampling. The first interview was conducted at the nearest household to the reference point if a respondent meeting the survey’s interests was available otherwise subsequent households were considered until the survey was concluded. The survey respondents’ categories included: (i) Men aged 15–49 years, (ii) Women aged 15–49 years, (iii) Male youth aged 15–24 years, (iv) Female youth aged 15–24 years, (v) Biological mothers with children aged 0–11 months, (vi) Biological mothers with children aged 12–23 months, (vii) Biological mothers with children aged 24–59 months, (viii) Orphans and vulnerable children 5–17 years. Only one respondent was interviewed per household and simple random sampling was used in the event there were more than respondents that met the criteria present. This approach ensured that the data were reliable and that it’s estimates were reliable to represent district performances moreover with comparable results to national surveys which are unreliable for district-based estimates [16]. This study involved the secondary analysis of a pooled dataset from the 2021 and 2022 LQAS surveys. Only eight DREAMS districts (Agago, Gulu, Omoro, Oyam, Apac, Kwania, Lira, and Mbarara) implemented the LQAS survey in 2021 and 2022 [18,26].

### Study population

The DREAMS program implements interventions for AGYW from the age of 10–24 years with customized interventions in the ages categories of 10–14 years, 15–19 years, and 20–24 years. However, the LQAS surveys do not collect data from participants who are less than 15 years old, therefore, for this study abstracted data was collected from AGWY between 15–24 years old. Of the 77 districts that implemented the LQAS surveys in 2021 and 2022, only 8 districts were DREAMS districts. These were in the regions of Lango (Oyam, Apac, Kwania, and Lira), Acholi (Agago, Gulu and Omoro) and Southwest (Mbarara). Within each region, a commensurate number of non-DREAMS districts were randomly selected for performance comparison Lango. The regional mapping and district implementation for the LQAS survey that influenced comparison can be found here [18,26].

### Study variables and measurements

Table 1 below shows how the outcome indicators were measured. The DREAMS program indicators are related to empowerment of AGYW, HIV risk reduction, mobilization of communities for change and strengthening of families with social protection. Based on this, the study abstracted the list of 15 indicators that were related to the desired outcomes of DREAMS interventions. The indicators were related to marital status (this sourced for early marriage), currently in school (which sourced for increase education rates among girls), HIV prevention indicators (testing, disclosure, knowledge and partner testing) and sexual behavior (activity, condom use and sexual partners).

**Table 1:** Measurement of outcome indicators.

Indicator	Question	Question Disaggregation	Numerator/Denominator
Percentage of AGYW by marital status	Q104: What is your current marital status?	Single, No partner; Single, Non-regular partner; Single with regular partner; Married; Living together Widowed; Divorced/Separated	(Married + Living together)/ Total
Percentage of AGYW that had ever been married	Q406: Have you ever been married?	Yes No	Yes/ Total
Percentage of AGYW that are currently in school	Q105: Are you currently in school?	Yes No	Yes/ Total
Percentage of AGYW that were pregnant	Q601: Are you or your partner currently pregnant?	Yes No	Yes/ Total
Percentage of AGYW that know where to test for HIV	Q201a: Do you know the nearest place where you can be tested for HIV?	Yes No	Yes/ Total
Percentage of AGYW that know how MTCT occurs	Q207: When can HIV be transmitted from an infected mother to her child? [TICK AS MANY RESPONSES AS MENTIONED]	Q207a: During pregnancy; Q207b: During delivery; Q207c: During breastfeeding; Q207d: Don’t know	Three options/ Total
Percentage of AGYW that know 3 ways to prevent HIV transmission	Q209: What are the ways of reducing HIV transmission from an infected mother to her child? [TICK ALL RESPONSES MENTIONED]	Q209a: Delivery in the hands of a trained health worker Q209b: Mother using ARVs Q209c: Testing and receiving results for HIV Q209d: Prevention of malaria during pregnancy Q209e: By operating the mother (Caeserian Section) Q209f: STI screening, prevention and treatment Q209g: Attending ANC Q209h: Baby given ARV syrup Q209i: Supplementation with Vitamin A & deworming Q209j: Replacement feeding Q209k: Exclusive breast feeding for first six months Q209l: Don’t know	Any three selected options/ Total
Percentage of AGYW that have ever been tested for HIV	Q202: I do not want you to tell me the results of the test; but have you ever been tested for HIV?	Yes No	Yes/ Total
Percentage of AGYW that had a recent HIV test	Q203a: I do not want you to tell me the results of the test; but have you taken an HIV test within the past 12 months?	Yes No	Yes/ Total
Percentage of AGYW that disclosed their HIV status to partner	Q203d: I do not want you to tell me the results of the test; but did you disclose your HIV results to your partner?	Yes No	Yes/ Total
Percentage of AGYW that had an HIV test with a sexual partner	Q204a: Have you taken an HIV test and received the test results together with your partner within the past 12 months?	Yes No	Yes/ Total
Percentage of AGYW that have ever had sex	Q407: Have you ever had sexual intercourse?	Yes No	Yes/ Total
Percentage of AGYW that had sex before 15 years	Q408a: How old were you when you had your first sexual intercourse? [ENTER ‘88’ IF Can’t remember]	Yes No	Yes/ Total
Percentage of AGYW that are sexually active	Q408b: Have you had sexual intercourse in the past 12 months?	Yes No	Yes/ Total
Percentage of AGYW that have multiple sex partners	Q409b: In the last 12 months i.e. since ${month} last year, how many people did you have sexual intercourse with?	Open ended	Mentioned > 1/ Total
Percentage of AGYW that used a condom during last sexual encounter	Q412: In the last act of sexual intercourse with a person who is not your wife (husband) or someone you live with or a regular partner did you use a condom?	Yes No	Yes/ Total

### Data collection and processing

The data for the LQAS survey were collected through interviewer-administered surveys. Trained field teams, including supervisors, were deployed to conduct face-to-face interviews with respondents in sampled households. The interview with the AGYW were conducted in private settings to ensure confidentiality, especially for sensitive questions related to sexual behavior and HIV testing. Consent and ascent were obtained from the parents and the respondents respectively before conducting interviews, and participants (and or their guardians) had the option to decline any questions or terminate the interview. The questionnaires were designed in English but translated during interviews in local languages, as well as English, based on the respondents’ preference to ensure clarity and ease of understanding. Language translation was part of the field team’s training to facilitate effective communication. Interviewers received instructions to ensure no other individuals, including parents, were present during interviews with respondents to protect privacy and minimize bias in responses. Emancipated minors were interviewed in the same manner as adults with considerations for confidentiality and ethical standards. Further information on the LQAS procedures can be found here [16,18,23–25].

### Statistical analysis

Overall, the analysis was conducted using LQAS survey data from 16 districts: eight DREAMS districts and eight randomly selected non-DREAMS districts. In the analysis, the exposure variable was being in a DREAMS district, and the outcomes were the levels of HIV prevention indicators. Percentages were calculated for each indicator to describe the distribution of outcomes within DREAMS and non-DREAMS districts. Each indicator was analyzed independently to evaluate performance differences. Differences in percentages between the DREAMS and non-DREAMS districts were tested for each indicator separately using the Pearson Chi-square test at a significance level of 5%. The results for each indicator were analyzed and interpreted independently. Although the findings are presented together in a single table for ease of interpretation, each indicator’s statistical significance was calculated separately without adjustments for multiple comparisons, given the independent examination of each outcome.

The analysis focused on valid responses for each indicator, as not all questions were applicable to the entire sample due to the application of skip logic. For example, questions related to sexual behavior were asked only to respondents who reported being sexually active. Similarly, follow-up questions about recent HIV testing were only asked to those who had previously indicated they had been tested for HIV. To ensure clarity and accuracy, the total number of valid responses for each indicator (denominator) is reported, excluding missing data and non-applicable responses. For instance, the total number of respondents who answered the “recent HIV test” question was lower than the total sample size because this question was only applicable to those who had ever been tested for HIV.

### Ethical consideration

The study was a secondary analysis of the LQAS survey data, which is publicly available upon reasonable request at the participating districts or projects without any restrictions on its use. As such, the study did not require ethical review consideration or informed consent. However, the investigators sought and received permission to use the survey datasets from the United States Agency for International Development (USAID)/ Strategic Information Technical Support (SITES) Activity. The analysis used an anonymized dataset without any identifying information that could have been collected as part of the survey. Further details regarding the conduct of the LQAS study may be found in the LQAS reports [27–29]. To strengthen the rigour of the results, the findings were reported following the STROBE guidelines for reporting observational studies in epidemiology [30].

## Results

### Analysis profile

**Fig 1:**
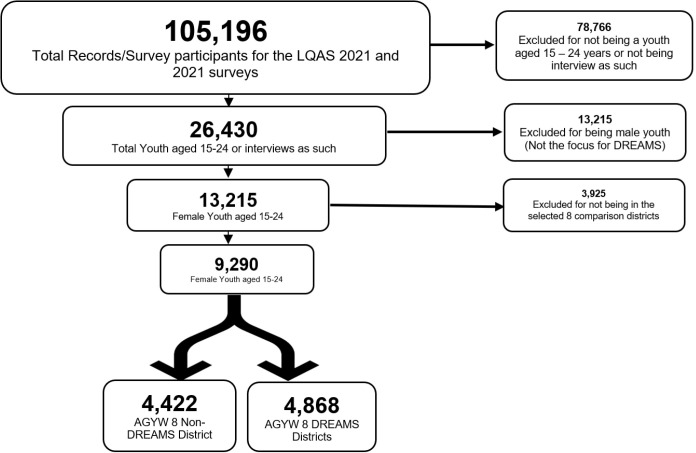
Study analysis profile.

The 2021 and 2020 LQAS survey datasets included pooled records of 105,196 participants. Of these, at total of 95,906 records (91.17%) were excluded from the analysis. 78,766 excluded records were of participants who were not youth aged 15–24 years. These were excluded because they were not interviewed as youth, implying that most data on HIV prevention indicators would be absent. Also, 13,215 records of male youth were excluded because they are not the primary focus for DREAMS programs. Finally, 3,925 records were excluded as these were not from the 8 selected comparison districts. Fig 1 shows the analysis profile for the study.

### Participant characteristics and comparative analysis of indicators between DREAMS and non-DREAMS districts

Table 2 below shows the findings of the comparative analysis of the level of HIV prevention levels between DREAMS and non-DREAMS districts. Overall, of 9,290 AGYW included in the analysis, the majority 62.24% were in the 20–24 year age group, married (71.52%), not in school (71.19%), knew where to test for HIV (92.93%), had ever had sex (78.99%), had not started sex before 15 years (89.6%), were sexually active (82.14%), did not have multiple sex partners (87.19%), used a condom during the last sexual encounter (53.91%), had ever been tested for HIV (83.5%), had a been tested recently for HIV (75.93%), had disclosed their HIV status to their partner (85.78%), were tested for HIV with their partner (79.91%), knew about mother-to-child transmission of HIV (84.38%), didn’t not know at least three ways of preventing HIV (79.62%), and were not pregnant (87.84%). Of the participants, 52.40% (4,868) resided in DREAMS districts while 47.60% (4,422) resided in non-DREAMS districts. Compared to non-DREAMS in DREAMS districts, statistically significant differences were observed in various indicators. A higher proportion of AGYW in DREAMS districts; were in school (30.26% versus 27.15%, *p* = 0.018), knew where to test for HIV (93.43% versus 92.38%, p = 0.049), had multiple sex partners (15.28% versus 10.11%, *p* < 0.001), used a condom at last sexual intercourse (58.99% versus 48.33%, *p* < 0.001), had ever been tested for HIV (84.86% versus 82%, *p* < 0.001), had a recent HIV test (77.60% versus 73.88%, *p* = 0.011), and knew at last three ways to prevent HIV (25.60% versus 14.63%, *p* < 0.001). Moreover, a lower proportion of AGWY in DREAMS districts were married compared to non-DREAMS districts (69.37% versus 73.88%, *p* < 0.001).

**Table 2:** Comparison of the level of HIV prevention indicators between DREAMS and non-DREAMS districts.

Indicator: *Percentage of AGYW that;*	Characterization	Total	Dreams district		*p-value*
			No	Yes	
General characteristics					
by marital status	Single	2646 (28.48)	1155 (26.12)	1491 (30.63)	*<0.001*
	Married	6644 (71.52)	3267 (73.88)	3377 (69.37)	
	*Total*	*9290 (100)*	*4422 (100)*	*4868 (100)*	
had ever been married	No	2488 (92.08)	1095 (93.11)	1393 (91.28)	*0.081*
	Yes	214 (7.92)	81 (6.89)	133 (8.72)	
	*Total* *	*2702 (100)*	*1176 (100)*	*1526 (100)*	
currently in school	No	3381 (71.19)	1618 (72.85)	1763 (69.74)	*0.018*
	Yes	1368 (28.81)	603 (27.15)	765 (30.26)	
	*Total* *	*4749 (100)*	*2221 (100)*	*2528 (100)*	
were pregnant	No	6330 (87.84)	3043 (87.12)	3287 (88.53)	*0.067*
	Yes	876 (12.16)	450 (12.88)	426 (11.47)	
	*Total* *	*7206 (100)*	*3493 (100)*	*3713 (100)*	
HIV knowledge					
know where to test for HIV	No	657 (7.07)	337 (7.62)	320 (6.57)	*0.049*
	Yes	8633 (92.93)	4085 (92.38)	4548 (93.43)	
	*Total*	*9290 (100)*	*4422 (100)*	*4868 (100)*	
know how MTCT occurs	No	1451 (15.62)	674 (15.24)	777 (15.96)	*0.34*
	Yes	7839 (84.38)	3748 (84.76)	4091 (84.04)	
	*Total*	*9290 (100)*	*4422 (100)*	*4868 (100)*	
know 3 ways to prevent HIV transmission	No	7397 (79.62)	3775 (85.37)	3622 (74.4)	*<0.001*
	Yes	1893 (20.38)	647 (14.63)	1246 (25.6)	
	*Total*	*9290 (100)*	*4422 (100)*	*4868 (100)*	
HIV testing					
have ever been tested for HIV	No	1533 (16.5)	796 (18)	737 (15.14)	*<0.001*
	Yes	7757 (83.5)	3626 (82)	4131 (84.86)	
	*Total*	*9290 (100)*	*4422 (100)*	*4868 (100)*	
had a recent HIV test	No	829 (24.07)	404 (26.12)	425 (22.4)	*0.011*
	Yes	2615 (75.93)	1143 (73.88)	1472 (77.6)	
	*Total* *	*3444 (100)*	*1547 (100)*	*1897 (100)*	
disclosed their HIV status to partner	No	293 (14.22)	151 (15.27)	142 (13.26)	*0.192*
	Yes	1767 (85.78)	838 (84.73)	929 (86.74)	
	*Total* *	*2060 (100)*	*989 (100)*	*1071 (100)*	
had an HIV test with a sexual partner	No	636 (20.09)	309 (20.67)	327 (19.57)	*0.441*
	Yes	2530 (79.91)	1186 (79.33)	1344 (80.43)	
	*Total* *	*3166 (100)*	*1495 (100)*	*1671 (100)*	
Sexual behavior					
have ever had sex	No	1378 (21.01)	615 (19.99)	763 (21.92)	*0.055*
	Yes	5180 (78.99)	2462 (80.01)	2718 (78.08)	
	*Total* *	*6558 (100)*	*3077 (100)*	*3481 (100)*	
had sex before 15 years	No	2549 (89.6)	1220 (89.12)	1329 (90.04)	*0.42*
	Yes	296 (10.4)	149 (10.88)	147 (9.96)	
	*Total* *	*2845 (100)*	*1369 (100)*	*1476 (100)*	
are sexually active	No	603 (17.86)	285 (17.7)	318 (18)	*0.823*
	Yes	2774 (82.14)	1325 (82.3)	1449 (82)	
	*Total* *	*3377 (100)*	*1610 (100)*	*1767 (100)*	
have multiple sex partners	No	2416 (87.19)	1191 (89.89)	1225 (84.72)	*<0.001*
	Yes	355 (12.81)	134 (10.11)	221 (15.28)	
	*Total* *	*2771 (100)*	*1325 (100)*	*1446 (100)*	
used a condom during last sexual encounter	No	1562 (46.09)	834 (51.67)	728 (41.01)	*<0.001*
	Yes	1827 (53.91)	780 (48.33)	1047 (58.99)	
	*Total* *	*3389 (100)*	*1614 (100)*	*1775 (100)*	

**Notes:** MTCT - Mother-to-child HIV transmission.

*The total (denominator) for each indicator may vary due to skip logics, please see Statistical Analysis section for details.

### Comparative analysis of indicator performance by age-group between DREAMS and non-DREAMS districts

Table 3 below shows the findings of the comparative analysis of level of indicators by age-group and DREAMS status. AGYW aged 15–19 years in DREAMS districts generally exhibited lower engagement in behaviors considered high risk compared to their peers in non-DREAMS districts. Specifically, marriage rates were lower (46.67% versus 52.82%, *p* < 0.001), sexual initiation lower (51.13% versus 55.71%, *p* = 0.020), condom usage was more frequent (54.36% versus 46.30%, *p* = 0.003), HIV testing (68.14% versus 62.89%, *p* = 0.001), and recent HIV testing was higher (72.84% versus 67.77%, *p* = 0.039). Disclosure of HIV status to partners and knowledge of HIV transmission were also higher among DREAMS participants (83.96% and 22.32%, respectively) compared to non-DREAMS (77.24% and 11.22%), *p* = 0.040 and *p* = 0.001 respectively. However, the proportion reporting multiple sexual partners was higher in DREAMS districts (18.63% versus 11.93%, *p* = 0.003).

**Table 3:** Comparison of indicators by age-group and DREAMS-district status.

Indicator: *Percentage of AGYW that;*	Disaggregation	AGYW between 15–19 years				AGYW between 20–24 years			
		Total	DREAMS district		P-value	Total	DREAMS district		P-value
			No	Yes		No	Yes		
General characteristics									
by marital status	Single	1764 (50.29)	820 (47.18)	944 (53.33)	<0.001	882 (15.25)	335 (12.48)	547 (17.66)	<0.001
	Married	1744 (49.71)	918 (52.82)	826 (46.67)		4900 (84.75)	2349 (87.52)	2551 (82.34)	
	Total	3508 (100)	1738 (100)	1770 (100)		5782 (100)	2684 (100)	3098 (100)	
had ever been married	No	1862 (95.68)	878 (96.27)	984 (95.16)	0.230	626 (82.8)	217 (82.2)	409 (83.13)	0.746
	Yes	84 (4.32)	34 (3.73)	50 (4.84)		130 (17.2)	47 (17.8)	83 (16.87)	
	Total *	1946 (100)	912 (100)	1034 (100)		756 (100)	264 (100)	492 (100)	
currently in school	No	1345 (53.67)	679 (55.61)	666 (51.83)	0.058	2036 (90.77)	939 (93.9)	1097 (88.25)	<0.001
	Yes	1161 (46.33)	542 (44.39)	619 (48.17)		207 (9.23)	61 (6.1)	146 (11.75)	
	Total *	2506 (100)	1221 (100)	1285 (100)		2243 (100)	1000 (100)	1243 (100)	
were pregnant	No	1822 (88.23)	940 (87.6)	882 (88.91)	0.357	4508 (87.69)	2103 (86.9)	2405 (88.39)	0.106
	Yes	243 (11.77)	133 (12.4)	110 (11.09)		633 (12.31)	317 (13.1)	316 (11.61)	
	Total *	2065 (100)	1073 (100)	992 (100)		5141 (100)	2420 (100)	2721 (100)	
HIV knowledge									
know where to test for HIV	No	436 (12.43)	232 (13.35)	204 (11.53)	0.101	221 (3.82)	105 (3.91)	116 (3.74)	0.75
	Yes	3072 (87.57)	1506 (86.65)	1566 (88.47)		5561 (96.18)	2579 (96.09)	2982 (96.26)	
	Total	3508 (100)	1738 (100)	1770 (100)		5782 (100)	2684 (100)	3098 (100)	
know how MTCT occurs	No	809 (23.06)	377 (21.69)	432 (24.41)	0.056	642 (11.1)	297 (11.07)	345 (11.14)	0.932
	Yes	2699 (76.94)	1361 (78.31)	1338 (75.59)		5140 (88.9)	2387 (88.93)	2753 (88.86)	
	Total	3508 (100)	1738 (100)	1770 (100)		5782 (100)	2684 (100)	3098 (100)	
know 3 ways to prevent HIV transmission	No	2918 (83.18)	1543 (88.78)	1375 (77.68)	<0.001	4479 (77.46)	2232 (83.16)	2247 (72.53)	<0.001
	Yes	590 (16.82)	195 (11.22)	395 (22.32)		1303 (22.54)	452 (16.84)	851 (27.47)	
	Total	3508 (100)	1738 (100)	1770 (100)		5782 (100)	2684 (100)	3098 (100)	
HIV testing									
have ever been tested for HIV	No	1209 (34.46)	645 (37.11)	564 (31.86)	0.001	324 (5.6)	151 (5.63)	173 (5.58)	0.945
	Yes	2299 (65.54)	1093 (62.89)	1206 (68.14)		5458 (94.4)	2533 (94.37)	2925 (94.42)	
	Total	3508 (100)	1738 (100)	1770 (100)		5782 (100)	2684 (100)	3098 (100)	
had a recent HIV test	No	409 (29.49)	205 (32.23)	204 (27.16)	0.039	420 (20.42)	199 (21.84)	221 (19.28)	0.153
	Yes	978 (70.51)	431 (67.77)	547 (72.84)		1637 (79.58)	712 (78.16)	925 (80.72)	
	Total *	1387 (100)	636 (100)	751 (100)		2057 (100)	911 (100)	1146 (100)	
disclosed their HIV status to partner	No	113 (19.38)	66 (22.76)	47 (16.04)	0.040	180 (12.19)	85 (12.16)	95 (12.21)	0.976
	Yes	470 (80.62)	224 (77.24)	246 (83.96)		1297 (87.81)	614 (87.84)	683 (87.79)	
	Total *	583 (100)	290 (100)	293 (100)		1477 (100)	699 (100)	778 (100)	
had an HIV test with a sexual partner	No	200 (22.3)	100 (22.08)	100 (22.52)	0.872	436 (19.22)	209 (20.06)	227 (18.5)	0.348
	Yes	697 (77.7)	353 (77.92)	344 (77.48)		1833 (80.78)	833 (79.94)	1000 (81.5)	
	Total *	897 (100)	453 (100)	444 (100)		2269 (100)	1042 (100)	1227 (100)	
Sexual behavior									
have ever had sex	No	1198 (46.65)	551 (44.29)	647 (48.87)	0.020	180 (4.51)	64 (3.49)	116 (5.38)	0.586
	Yes	1370 (53.35)	693 (55.71)	677 (51.13)		3810 (95.49)	1769 (96.51)	2041 (94.62)	
	Total *	2568 (100)	1244 (100)	1324 (100)		3990 (100)	1833 (100)	2157 (100)	
had sex before 15 years	No	877 (84.08)	453 (84.83)	424 (83.3)	0.500	1672 (92.79)	767 (91.86)	905 (93.59)	0.156
	Yes	166 (15.92)	81 (15.17)	85 (16.7)		130 (7.21)	68 (8.14)	62 (6.41)	
	Total *	1043 (100)	534 (100)	509 (100)		1802 (100)	835 (100)	967 (100)	
are sexually active	No	303 (23.04)	146 (21.66)	157 (24.49)	0.222	300 (14.55)	139 (14.85)	161 (14.3)	0.723
	Yes	1012 (76.96)	528 (78.34)	484 (75.51)		1762 (85.45)	797 (85.15)	965 (85.7)	
	Total *	1315 (100)	674 (100)	641 (100)		2062 (100)	936 (100)	1126 (100)	
have multiple sex partners	No	858 (84.87)	465 (88.07)	393 (81.37)	0.003	1558 (88.52)	726 (91.09)	832 (86.4)	0.002
	Yes	153 (15.13)	63 (11.93)	90 (18.63)		202 (11.48)	71 (8.91)	131 (13.6)	
	Total *	1011 (100)	528 (100)	483 (100)		1760 (100)	797 (100)	963 (100)	
used a condom during last sexual encounter	No	656 (49.77)	363 (53.7)	293 (45.64)	0.003	906 (43.75)	471 (50.21)	435 (38.39)	<0.001
	Yes	662 (50.23)	313 (46.3)	349 (54.36)		1165 (56.25)	467 (49.79)	698 (61.61)	
	Total *	1318 (100)	676 (100)	642 (100)		2071 (100)	938 (100)	1133 (100)	

**Notes:**

MTCT - Mother-to-child HIV transmission.

*The total (denominator) for each indicator may vary due to skip logics, please see Statistical Analysis section for details.

Among the AGYW aged 20–24 years, marriage rates were lower in DREAMS districts (82.34%) versus non-DREAMS (87.52%), *p*=<0.001 and school attendance was higher (11.75% versus 6.10%, *p* < 0.001). The proportion reporting multiple sexual partners was higher in DREAMS districts (13.60%) compared to non-DREAMS (8.91%), *p* = 0.002, while condom usage was higher (61.61% vs. 49.79%, *p* < 0.001). Knowledge of HIV transmission remained better in DREAMS districts (27.47%) compared to non-DREAMS (16.84%), *p* < 0.001.

## Discussion

This study assessed differences in HIV prevention indicator levels among AGYW in DREAMS and non-DREAMS districts to examine possible associations with the presence of DREAMS interventions. The findings indicated associations between residing in DREAMS districts and better HIV prevention indicator outcomes. AGYW in DREAMS districts were more likely to be enrolled in school, less likely to be married, aware of HIV testing services, knowledgeable about HIV prevention, and reported higher rates of HIV testing and condom use. However, a higher proportion of AGYW in DREAMS districts also reported having multiple sexual partners. Similar observations were made in age group comparisons.

AGYW in DREAMS districts had better levels of various HIV prevention indicators outcomes compared to their counterparts in non-DREAMS districts. Studies in South Africa and Kenya have found similar findings [5–7]. Such associations are linked to reduced HIV prevalence among AGYW [4,14,15] and align with expected outcomes if DREAMS interventions are functioning as designed. For instance later marriage can reduce HIV due to improve negotiating power for safe sex [14,31,32], higher condom use and HIV testing rates highlight better access to HIV prevention services [33] while disclosure and improved knowledge suggest greater awareness and communication around HIV prevention [7,34,35]. However, within DREAMS districts, DREAMS recipients were not specifically selected for the original survey whose data are used in this study. Therefore, the findings could suggest the potential for a catalytic effect, possibly due to peer-to-peer interactions and community-wide behavioral shifts [36]. DREAMS interventions provide critical prevention packages for AGYW, but scalability remains a challenge due to financial constraints. If catalytic effects are contributing to community-wide improvements, they could provide a more practical model for scale-up, warranting further exploration [37]. Nonetheless, the differences could be due to underlying contextual factors specific to DREAMS districts, rather than direct effects of the interventions.

The finding that a higher proportion of AGYW in DREAMS districts reported having multiple sexual partners may suggest a persistent behavioral challenge in DREAMS districts. This result appears counterintuitive, especially considering this same study also found higher levels of HIV knowledge and prevention aspects in the districts. While this pattern could partly explain why these districts were selected for DREAMS interventions, it also suggests that certain risk behaviors persist despite program efforts. The Uganda Population-Based HIV Impact Assessment (UPHIA) findings also showed that AGYW in DREAMS regions had the highest rates of non-marital/non-cohabiting sexual engagement (43%) with similar rates in across DREAMS regions [20]. Multiple sex partners increase the risk of acquiring HIV and HIV transmissions [38–40]. This may undermine the contributory effect of other DREAMS interventions on declining trends in new HIV infections [41]. Moreover, there has been an increase in HIV prevalence from 5.4% in 2016/17 to 7.6% in 2020/21 in the Mid North [2,20] (where seven of the eight DREAMS districts are located, and where the DREAMS program has been implemented for the longest period). This underscores the complexity of addressing HIV risk factors in these communities and may call for more refined interventions that target number of sexual partners by AGYW in DREAMS districts.

This study was a secondary analysis of district-based Lot Quality Assurance Sampling (LQAS) survey data. Although unweighted data were used, potentially leading to slight over- or underestimates, previous studies suggest minimal differences between crude and weighted estimates [42–44]. Therefore, the findings are considered reliable but should be interpreted with caution. A key limitation is the non-random selection of DREAMS districts, chosen based on higher HIV risk. This likely led to baseline differences between DREAMS and non-DREAMS districts, making it difficult to determine whether observed differences are due to DREAMS interventions or pre-existing contextual factors. The absence of baseline data further complicates the comparability of these districts over time, posing challenges in attributing the observed differences solely to DREAMS implementation. Future research could use this study as a baseline for comparison, employ more rigorous methods that can help reduce selection bias and provide more accurate estimates of program influence. Not all AGYW in DREAMS districts received interventions, suggesting that broader community-level effects or peer-to-peer interactions may have influenced the findings [37]. The cross-sectional design and interview-administered questionnaires employed by the initial survey may have introduced limitations common to such designs, including social desirability bias [45,46]. Moreover, as a secondary analysis, this study was limited to the variables and data available from the original survey, restricting the ability to explore additional factors or potential confounders [47].

## Conclusions

This study found associations between residence in DREAMS districts and more favorable level of HIV prevention indicators outcomes among AGYW in Uganda. AGYW in DREAMS districts reported lower early marriage rates, increased HIV testing, higher condom use, and improved HIV prevention knowledge compared to their counterparts in non-DREAMS districts. However, a persistent gap remains regarding higher rates of multiple sexual partners among AGYW. This behavioral challenge could undermine the potential benefits of other positive indicators. Increasing focus on interventions regarding reducing the number of sexual partners, girl protection and negotiation skills could help reduce risky behavior. However, it is also worth considering that part of the results may support a greater emphasis on peer-to-peer influence and community-wide behavior change strategies to amplify positive behavioral shifts. This suggests the potential for developing a scale-up framework where strategically targeting specific districts or sub-counties could achieve broader national HIV prevention goals without the need for comprehensive coverage, however, this warrants further analytical considerations. Finally, it is crucial to interpret these findings as associations rather than causal impacts, given the cross-sectional design and potential selection bias due to the non-random allocation of DREAMS districts. Future research using more rigorous study designs and statistical approaches is recommended to strengthen causal inferences.
